# Social Isolation and Memory Decline in Later-life

**DOI:** 10.1093/geronb/gbz152

**Published:** 2019-11-29

**Authors:** Sanna Read, Adelina Comas-Herrera, Emily Grundy

**Affiliations:** 1 Care Policy and Evaluation Centre, London School of Economics and Political Science, UK; 2 Institute for Social and Economic Research, University of Essex, Colchester, UK

**Keywords:** Cognition, Longitudinal methods, Social networks, Structural equation models

## Abstract

**Objectives:**

To investigate associations between level and changes in social isolation and in memory in older men and women.

**Methods:**

The sample included 6,123 women and 5,110 men aged 50+ from the English Longitudinal Study of Aging (ELSA). Extended latent change score models from six measurement occasions every 2 years from 2002 were used to investigate associations between social isolation and memory. Models were adjusted for age, socioeconomic position, and health.

**Results:**

Social isolation increased and memory decreased over time. Among men an initially high level of social isolation was associated with a somewhat greater decrease in memory. Among women a greater increase in social isolation predicted a greater decrease in memory and a larger change in social isolation was associated with further larger changes in isolation, although when social isolation reached a higher level it subsequently decreased.

**Conclusions:**

Results suggest that the association between social isolation and memory decline arises because social isolation is associated with increased memory decline rather than poor memory leading to increases in social isolation. Men with high levels of social isolation and women with accumulated social isolation over time are especially affected as these patterns of isolation were associated with more profound memory decline.

Memory, the ability to retrieve information from the past, tends to decline over the second half of the life course, and memory decline is one of the greatest worries older people have about aging (Molden & Maxfield, 2017). Understanding pathways to changes in memory is thus important, especially in the context of population aging. Due to losses in social relationships (Bowling, Grundy, & Farquhar, 1995), risks of social isolation, defined as low diversity and frequency of social contacts, may also increase in later life and may contribute to increasing memory problems (Fratiglioni, Wang, Ericsson, Maytan, & Winblad, 2000). However, social isolation has also been considered as a potential outcome of poor memory (Thomas, 2011). To disentangle the direction of these hypothesized pathways, we investigate how both levels and changes in social isolation and memory are associated with subsequent changes using data from six waves of The English Longitudinal Study of Aging (ELSA).

Social isolation has numerous detrimental health effects, including higher morbidity and mortality (for recent reviews, see Courtin & Knapp, 2017; Leigh-Hunt et al., 2017). A number of studies also indicate that perceived isolation and quality of social contacts play an important role in cognitive functioning (Cacioppo, Hawkley, & Hawkey, 2009; Gow, Corley, Starr, & Deary, 2013; O’Luanaigh et al., 2012; Wilson et al., 2007). Other studies which have used quantitative measures of social isolation (e.g., number and/or frequency of social interactions) have found associations with various dimensions of cognition, especially poor memory and dementia in later life (Bennett, Schneider, Tang, Arnold, & Wilson, 2006; DiNapoli, Wu, & Scogin, 2014; Evans, Llewellyn, et al., 2018; Fratiglioni et al., 2000; Shankar, Hamer, McMunn, & Steptoe, 2013). Two recent reviews have also suggested an association between social isolation or low levels of social contacts with cognitive functioning (Evans, Martyr, Collins, Brayne, & Clare, 2018) and cognitive decline (Kuiper et al., 2016).

The mechanisms that link social isolation with poor cognition may include the detrimental effect of a lack of social stimulation on the brain which may result in lower cognitive reserve, poorer resilience of the brain, and cognitive decline (Evans, Llewellyn, et al., 2018). Alternatively, lower cognitive reserve among people with few social relationships (Bennett et al., 2006), and further cognitive decline may precede withdrawal from social interactions. Apart from the lack of social stimulation, social isolation may also induce prolonged stress that in turn may reduce cognitive functioning (Seeman, Lusignolo, Albert, & Berkman, 2001). Furthermore, it may be difficult to differentiate between effects of social isolation itself and conditions such as depression and perceived loneliness (dissatisfaction with the frequency and closeness of contacts), all of which may be associated with cognitive function and physical health (Luo, Hawkley, Waite, & Cacioppo, 2012; Shankar et al., 2013).

To unravel the mechanisms underlying associations between social isolation and cognitive function, there is a need to establish the direction of effects and the interrelationships between levels, changes, and trajectories of change in both social isolation and cognitive function. Previous studies have noted a dearth of research on these processes (Courtin & Knapp, 2017; Sörman, Rönnlund, Sundström, Adolfsson, & Nilsson, 2015). Although there have been some studies of longitudinal associations between social isolation and cognition, they usually focus on the effect of initial social isolation on changes in cognition at follow-up. Andrew and Rockwood (2010), for example, reported that social vulnerability, measured using a wide range of both quantitative and qualitative aspects of social engagement (including living situation, marital status, social support, and feelings of mastery and empowerment), was associated with cognitive decline over a 5-year follow-up. Another study found that social vulnerability score predicted cognitive decline at 3- and 6-year follow-ups in the Honolulu–Asia Aging Study (Armstrong et al., 2015). Shankar and colleagues (2013), in a 4-year follow-up of participants in the ELSA, also reported that social isolation, but not loneliness, was associated with cognitive decline.

These studies assumed that social isolation predicts cognitive decline and did not test for an alternative direction of effects. However, people with poor or deteriorating cognition, including memory problems, may withdraw from social interaction and become more isolated (Thomas, 2011). It is also possible that families of older people with memory problems respond by initiating more contacts, with a resulting decrease in social isolation (Kotwal, Kim, Waite, & Dale, 2016). Using the Americans’ Changing Lives Survey, Thomas (2011) assessed cross-lagged associations between social engagement (defined as frequency of social activities such as phoning or visiting friends and family, attending groups/organizations/religious services, and volunteering), and cognition to determine the direction of effects over time. Results showed that in women greater social engagement predicted higher cognition, whereas in men, lower cognition predicted lower social engagement, suggesting that women may benefit more from social engagement whereas in men associations may be more affected by selection. This study suggested the direction of associations between levels of social engagement and cognition, but could not establish directions of effects related to changes over time. In another study, Thomas (2012) created five latent classes of social engagement of which the one showing low initial level and decline over 16 years was associated with faster cognitive decline. This study analyzed changes over time but could not disentangle the order between the processes.

Some studies have found no association between social isolation and cognitive change: Green and colleagues (2008) found no association between network size (number of relatives, friends, and neighbors the respondent had contacts with during the last 6 months) or frequency of these contacts and memory (delayed recall) in a 10-year follow-up of older people in Baltimore in the United States. Holwerda and colleagues (2014) reported no association between social isolation and onset of dementia in a sample of older people in the Netherlands. In a 6-year follow-up among older English people, being not married and having a low number of close contacts, but not the overall frequency of contacts with children, other relatives and friends or participation in organizations, were associated with onset of dementia (Rafnsson, Orrell, Orsi, Hogervorst, & Steptoe, 2017). However, these studies only included social contact measures at baseline and could not assess the direction of associations between changes in social contacts and cognition. Moreover, possible gender differences were not examined.

Although several of the studies referred to above (Andrew & Rockwood, 2010; Armstrong et al., 2015; Shankar et al., 2013; Thomas, 2011, 2012) lend support to the hypothesis of a link between social isolation and cognition or cognitive change, none of these studies assessed associations between the initial level of either factor and subsequent change. To fill this gap, in this study we analyze the effects of level-to-change and also change-to-change (trajectories). We test whether the level of social isolation, low diversity, and frequency of contacts, is associated with increases in memory decline over time or whether alternatively poorer memory increases social isolation in later life. The modeling strategy adopted also makes it possible to test whether an *increase* (change) in social isolation predicts a decrease in memory or vice versa. Previous research has shown that patterns and trajectories of social interaction vary substantially by gender in older age groups, for example, older women more frequently live alone compared to men but have higher levels of engagement in other social activities and more social contacts (Milligan, Payne, Bingley, & Cockshott, 2015). Cognition also varies by gender with dementia being more frequent among women (Mayeda, 2019). Additionally, as discussed above, previous studies suggest gender differences in directions of association between social isolation and cognition (Thomas, 2011). For these reasons, we undertook separate analyses for men and women.

## Method

### Data

We used a sample of men and women from the ELSA, a nationally representative longitudinal study of the older population of England (Steptoe, Breeze, Banks, & Nazroo, 2013). The first wave of ELSA, conducted in 2002–2003, included men and women then aged 50 years or more from private households which had participated in any one of the 1998, 1999, or 2001 rounds of the cross-sectional Health Survey for England (HSE); an annual government health survey based on a stratified random sample of all households in England. Response rates for the HSE were 69% in 1998, 70% in 1999, and 67% in 2001. A total of 11,392 core members were interviewed in the first wave of ELSA (response rate 67%; for more information on response rates and nonresponse, see Bridges, Hussey, & Blake, 2015; Steptoe et al., 2013). Comparisons with other sources, including the national census, showed that the baseline ELSA was nationally representative (Marmot, Banks, Blundell, Lessof, & Nazroo, 2003). Respondents have been reinterviewed every 2 years. Although those in institutional settings were not included in the initial sample, sample members who moved to institutional settings during the follow-up period have been retained in the study. Participants gave their informed consent to take part in the study. Ethical approval was given by the London Multicentre Research Ethics Committee.

Information from Waves 1 to 6 was used. The analysis sample included 11,233 participants in Wave 1. Numbers of respondents with data available for different measures are given in Supplementary Tables 1–3. In wave 1, respondents with available data for different variables used in the analysis ranged between 4,650 and 5,110 in men and 5,532 and 6,123 in women. In Wave 6, data on repeated measures were available for 42%–48% of men and from 44% to 52% of women who participated in Wave 1.

### Measures

*Memory* was tested with a word list recall in which the participant was asked to learn 10 common unrelated words (Hubbert, Gardener, & McWilliams (2006). Mean score for immediate recall and for delayed recall was used in the analysis (Cronbach’s alpha = 0.82–0.86 in the six waves). The mean score was normally distributed.

*Social isolation* was measured using an index derived from five binary items: whether (a) the respondent lived alone; (b) had less than monthly contact including face-to-face, telephone, or written/email contact with child(ren), (c) other family members or (d) friends; and (e) if they were not a member of any organizations, religious groups or committees (de Oliveira, Shankar, Kumari, Nunn, & Steptoe, 2010; Shankar et al., 2013). The score ranged from 0 to 5 (number of “yes” answers to the five items above), with higher scores indicating greater social isolation. The distribution was somewhat skewed but was treated as continuous in the models using maximum likelihood estimation with robust standard errors (MLR) which can handle nonnormality.

### Covariates

Indicators of socioeconomic status (education, wealth, home ownership), and health-related behaviors (smoking, physical activity) changed very little over time so were treated as time-invariant using values from Wave 1. Age was also measured at baseline. Limiting long-term illness, depressive symptoms, and whether working or doing voluntary work were treated as time-varying covariates. These covariates were included because they are known to be associated both with social isolation and with poorer cognition, including memory (Agrigoroaei & Lachman, 2011; Bielak, Hughes, Small, & Dixon, 2007; Seeman et al., 2001; Shankar, McMunn, Banks, & Steptoe, 2011; Thomas, 2011; Toepoel, 2013).

Age (single years) was treated as a continuous measure. Educational level indicated respondents’ highest qualification: tertiary level (college or university diploma or degree) used as the reference group; upper secondary (O′ or A′ levels or equivalent public examinations taken in secondary schools at around ages 16 and 18, respectively); other (e.g., vocational or foreign qualifications); or no or lower level qualifications. Wealth quintiles were calculated using nonpension wealth indicating financial, physical, and housing wealth net of debt. Wealth quintile was treated as continuous in the analysis. Home ownership was a binary measure, 1 indicating home owning outright, through mortgage or shared-ownership, and 0 renting, living rent free or squatting.

Smoking was measured with three binary variables distinguishing current smokers, ex-smokers, and never smokers. Self-reported physical activity included four categories: sedentary (no physical activity and/or a sedentary job), low (mild physical activity at least once a week and/or in a job that was mostly standing), moderate (moderate physical activity at least once a week and/or in a job involving physical work), and high (vigorous physical activity at least once a week and/or in a job involving heavy manual labor; de Oliveira et al., 2010). Because the distribution of physical activity was approximately normal and the association with outcomes linear, this measure was treated as continuous.

We used a binary variable indicating whether the respondent reported any limiting long-term illness (yes/no). Depressive symptoms were measured with a short version of the Centre for Epidemiological Studies Depression Scale (CES-D; Radloff, 1977). The scale included eight binary items so that the count of depressive symptoms ranged from 0 to 8. Because of the skewed distribution we dichotomized this variable (three or more symptoms coded as 1, zero to two symptoms as 0). A binary measure of working or voluntary work in the past month (yes/no) included paid or voluntary work, self-employment or work-related training.

### Analysis

We used an extended latent change score (LCS) model (Grimm, An, McArdle, Zonderman, & Resnick, 2012; McArdle, 2009; McArdle & Hamagami, 2001) based on the structural equation framework to investigate the direction of longitudinal associations between social isolation and memory. The model includes a latent growth curve model part, which in the present study assessed intercept, linear change, and proportional change (curvature or acceleration of change). The model estimates a latent difference score between each measurement occasion, which can be used to assess the direction of associations, for example, whether the level of social isolation predicts faster decline in memory or whether poorer level of memory predicts increased social isolation. Moreover, the extended part of the model enables assessment of bivariate effects of a recent change on subsequent change (change-to-change; Grimm et al., 2012), for example, whether a faster increase in social isolation predicts a faster decline in memory or vice versa. The change-to-change parameters are added in addition to the usual level-to-change components in the LCS. They allow measurement of the effect of the previous change on the subsequent change within a variable (univariate) and/or between the variables (bivariate).

Analyses were carried out using Mplus software version 7.3 (Muthén & Muthén, 1998–2015). Measurements collected at six time points were used to estimate the initial level, linear change, and acceleration in social isolation and memory. Models were adjusted for age, education, wealth, home ownership, smoking, physical activity, limiting long-term illness, depressive symptoms, and working or doing voluntary work. Continuous covariates were centered to make the interpretation of the estimates easier.

The nested models were compared using the likelihood ratio test which indicates the change in −2 log likelihood (−2LL) with respect to the change in the number of parameters in the model. This model comparison makes it possible to decide whether parameters are necessary (or can be set to 0). A significant *p*-value in the likelihood ratio test indicates that the term is needed in the model. Akaike’s information criterion (AIC) and the Bayesian information criterion (BIC) were also used to compare different models: a lower AIC/BIC value indicates a better model fit. Model comparisons were started from the full-saturated model including all parameters. Parameters were dropped one at a time and fit was compared with the previous model. The full model included all paths to control for the effect of all dynamic parameters.

Maximum likelihood estimation with robust standard errors (MLR) was used to take into account any nonnormality in the sample. Full information maximum likelihood (FIML) was used. This method includes all respondents in the data regardless of whether they participated in later waves or responded to all items. The approach uses all available information on mean and variance of variables.

## Results

### Descriptive Results

Descriptive results for the time-invariant variables from Wave 1 are given in Supplementary Table 1. The average age of respondents was 65. About a third of men and nearly half of women had no educational qualifications, whereas 28% of men and 17% women had tertiary education. Compared with men, a higher proportion of women were in lower wealth quintiles. About 80% of participants were homeowners. Fewer than 20% of either men and women were current smokers and most respondents reported moderate physical activity.

The time-varying covariates are given in Supplementary Table 2 for men and Supplementary Table 3 for women. Most people reported at least one item on the social isolation scale ranging from zero to five. In the memory tests, on average five word list items were retrieved immediately and four items after a delay. Memory score was between 4.2 and 4.9 and tended to decline somewhat over the follow-up. A higher proportion of women than men reported three or more depressive symptoms (about 27% vs about 21%). Limiting long-term illness was reported by about a third of both men and women at baseline and by nearly half at the end of the follow-up. The proportions working or doing voluntary work dropped from 49% to 26% in men and 42% to 21% in women over the follow-up period.

Those who did not provide complete information in one or more of the five follow-ups were older, had lower socioeconomic status, less advantageous health behaviors, poorer health, higher social isolation, and lower memory score at the Wave 1 baseline, compared with those who completed all six waves (results available from the authors on request).

### Univariate LCS Models

Supplementary Table 4 shows the parameter estimates for social isolation and memory in men and women in the univariate LCS models. As a group, women showed a slight overall decline in memory, on average a decline of one point on the memory scale over 10 years, corresponding to a 3.7% decline in memory over 2 years. Women also showed an overall increase in social isolation (on average 0.4 points over 10 years). There were interindividual differences in the level of memory and social isolation and in change over time in memory. Individual variation around change in social isolation suggested similarity of participants’ trajectories over time. Women who had experienced more change in social isolation were likely to experience more change in the future. This accumulation was somewhat reversed by the effect of the isolation level so that when isolation reached a higher level it decreased in the next time window. Overall, an initial increase of one point in the social isolation scale resulted in another increase of about one point over the 10-year period, when taking into account the reversing effect of the higher level of isolation. Previous decline in memory also predicted future decline in memory in women.

In men, neither memory nor social isolation showed any proportional change (the effect of level on change) or change on change (the effect of previous change on subsequent change; Supplementary Table 4).

Of the covariates in Supplementary Table 4, older age was associated with a higher level of social isolation and lower memory score. Older age amplified the increase in social isolation. Lower education was associated with poorer memory score. In men, lower educational level was also to some extent associated with social isolation. Lower wealth quintile and not being a homeowner were associated with social isolation and poorer memory score. Higher wealth quintile was also associated with a smaller increase in social isolation over time in men. Current smoking was associated with a higher level and faster increase in social isolation in women. Those having a high level of physical activity reported a lower level of social isolation and had a higher memory score. All time-varying covariates—limiting long-term illness, depressive symptoms, and working or doing voluntary work—were associated with social isolation and memory to some extent. It is important to note that all covariates were entered into the model simultaneously, so results are fully adjusted. Thus, for instance, adjusting for socioeconomic status and health-related behaviors may partly overlap with the effects of limiting long-term illness, depressive mood, and working/voluntary work, in which case each variable’s independent effect may be reduced in the fully adjusted model.

### Bivariate LCS Models

**Figure 1.**
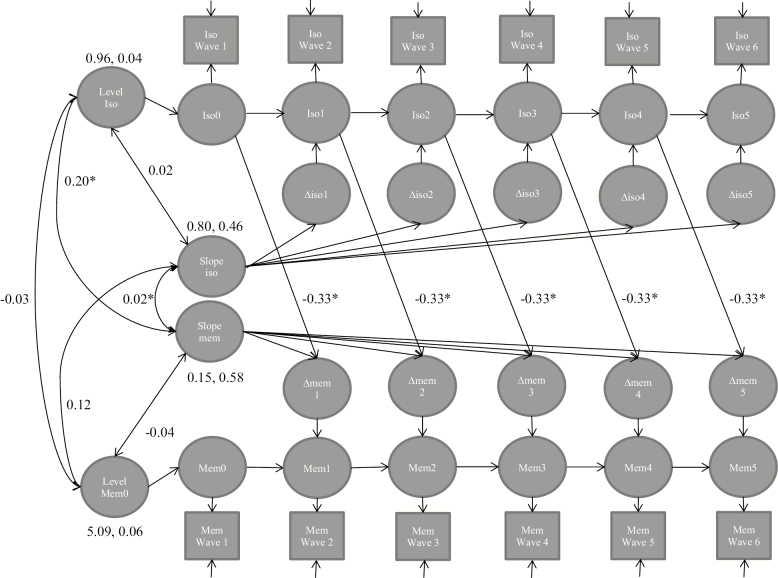
Full bivariate latent change score model for men (*N* = 5,110). The diagram shows that the level of social isolation predicts the change in memory over the six waves. Only significant level-to-change paths are shown (see Table 1 for the estimates of all paths), paths with no coefficient are fixed to 1, fully adjusted models. The model fit: −2LL = 140,599.01, *df* = 469, AIC = 282,136, BIC = 285,202. Iso = social isolation, mem = memory. *p < .05, **p < .01, ***p < .001.

Table 1 shows the results from the bivariate LCS model estimates for the parameters in the full models estimating how initial level predicts change (level-to-change model) and the extended model of how previous change predicts subsequent change (change-to-change model). In men, no associations between previous and subsequent changes were found in the extended models (Tables 1 and 2). The full level-to-change model (Table 1, Figure 1) showed a modest association between social isolation and change in memory: men with a higher level of social isolation experienced a faster decline in memory. The model comparisons (level-to-change model 3 for men, Table 2) suggested that this path was necessary in the model and cannot be set to 0. A high social isolation score (4+) was associated with a memory decline of 0.9 points (18%) over 2 years, compared with a 0.3-point memory decline (6%) among those men with the average isolation score of one.

**Table 1.** Parameter Estimates (Standard Error, *SE*) for the Full Bivariate Latent Change Score Models^a^ Waves 1–6 in ELSA

	Men (*N* = 5,110)	Women (*N* = 6,123)
Latent change score model	Est. (*SE*)	Est. (*SE*)
Level-to-change model paths^b^		
Level social isolation → Change social isolation	−0.09 (0.11)	−0.07 (0.06)
Level memory -> Change memory	0.02 (0.10)	0.15 (0.11)
Level social isolation → Change memory	−0.33 (0.15)*	−0.08 (0.16)
Level memory → Change social isolation	−0.14 (0.09)	−0.11 (0.06)
Change-to-change model paths^c^		
Level social isolation → Change social isolation	−0.17 (0.16)	−0.37 (0.12)**
Level memory → Change memory	−0.50 (0.49)	0.03 (0.19)
Level social isolation → Change memory	0.07 (0.33)	0.44 (0.25)
Level memory → Change social isolation	−0.16 (0.18)	0.00 (0.08)
Change social isolation → Change social isolation	−0.03 (0.36)	0.97 (0.38)*
Change memory → Change memory	1.55 (1.14)	−0.17 (0.49)
Change social isolation → Change memory	−0.78 (1.13)	−2.61 (0.75)**
Change memory → Change social isolation	0.12 (0.48)	−0.23 (0.29)

*Note.* −2LL = −2 log likelihood, *df* = degrees of freedom, AIC = Akaike’s information criterion, BIC = Bayesian information criterion.

^a^Adjusted for age, education, net wealth quintile, tenure status, smoking, and physical activity from Wave 1 and time-varying variables of limiting long-term illness, depression, and working/doing voluntary work from Waves 1 to 6.

^b^The full level-to-change model fit: −2LL = 140,599.01, *df* = 469, AIC = 282,136, BIC = 285,202 for men; −2LL = 176,112.58, *df* = 469, AIC = 353,163, BIC = 356,315 for women.

^c^The full change-to-change model fit: −2LL = 140,596.49, *df* = 473, AIC = 282,139, BIC = 285,231 for men; −2LL = 176,095.99, *df* = 473, AIC = 353,138, BIC = 356,316 for women.

*p < .05. **p < .01. ***p < .001.

**Table 2.** Comparison of the Paths in the Latent Change Score Models^a^ Waves 1–6 in ELSA

	Men (*N* = 5,110)				Women (*N* = 6,123)			
Latent change score model	Model comp.	∆−2LL (*df*)	AIC	BIC	Model comp.	∆−2LL (*df*)	AIC	BIC
Level-to-change models: paths set to 0								
1. Level social isolation → Change social isolation	0^b^ vs 1	3.12 (1) ^b^	282,140^b^	285,200^b^	0^c^ vs 1	1.24 (1)^c^	353,164^c^	356,309^c^
2. Level memory → Change memory	1 vs 2	0.00 (1)	282,138	285,192	1 vs 2	1.88 (1)	353,165	356,304
3. Level social isolation → Change memory	2 vs 3	4.16 (1)*	282,144	285,192	2 vs 3	3.34 (1)	353,170	356,302
4. Level memory → Change social isolation	2 vs 4	0.79 (1)	282,137	285,185	3 vs 4	2.93 (1)	353,174	356,299
Change-to-change models: paths set to 0								
1. Level social isolation → Change social isolation	0^d^ vs 1	0.86 (1)^d^	282,139^d^	285,225^d^	0^e^ vs 1	8.26 (1)** ^,e^	353,152^e^	356,324 ^e^
2. Level memory → Change memory	1 vs 2	1.71 (1)	282,140	285,220	2 vs 0^e^	0.01 (1)	353,136	356,308
3. Level social isolation → Change memory	2 vs 3	1.97 (1)	282,142	285,215	2 vs 3	2.81 (1)	353,140	356,305
4. Level memory → Change social isolation	3 vs 4	1.62 (1)	282,143	285,210	3 vs 4	0.19 (1)	353,138	356,296
5. Change social isolation → Change social isolation	4 vs 5	0.16 (1)	282,141	285,202	4 vs 5	6.60 (1)*	353,149	356,301
6. Change memory → Change memory	5 vs 6	1.03 (1)	282,142	285,195	4 vs 6	1.42 (1)	353,139	356,290
7. Change social isolation → Change memory	6 vs 7	3.09 (1)	282,144	285,193	6 vs 7	9.96 (1)**	353,157	356,302
8. Change memory → Change social isolation	7 vs 8	0.16 (1)	282,144	285,185	6 vs 8	0.09 (1)	353,148	356,292

*Note.* ∆−2LL (*df*) = difference in −2 log likelihood and degrees of freedom between the nested models, AIC = Akaike’s information criterion, BIC = Bayesian information criterion.

^a^Adjusted for age, education, net wealth quintile, tenure status, smoking, and physical activity from Wave 1 and time-varying variables of limiting long-term illness, depression, and working/doing voluntary work from Waves 1–6.

^b^Comparison to the full level-to-change model for men: −2LL = 140,599.01, *df* = 469, AIC = 282,136, BIC = 285,202.

^c^Comparison to the full level-to-change model for women: −2LL = 176,112.58, *df* = 469, AIC = 353,163, BIC = 356,315.

^d^Comparison to the full change-to-change model for men: −2LL = 140,596.49, *df* = 473, AIC = 282,139, BIC = 285,231.

^e^Comparison to the full change-to-change model for women: −2LL = 176,095.99, *df* = 473, AIC = 353,138, BIC = 356,316.

*p < .05. ** p < .01. ***p < .001.

**Figure 2.**
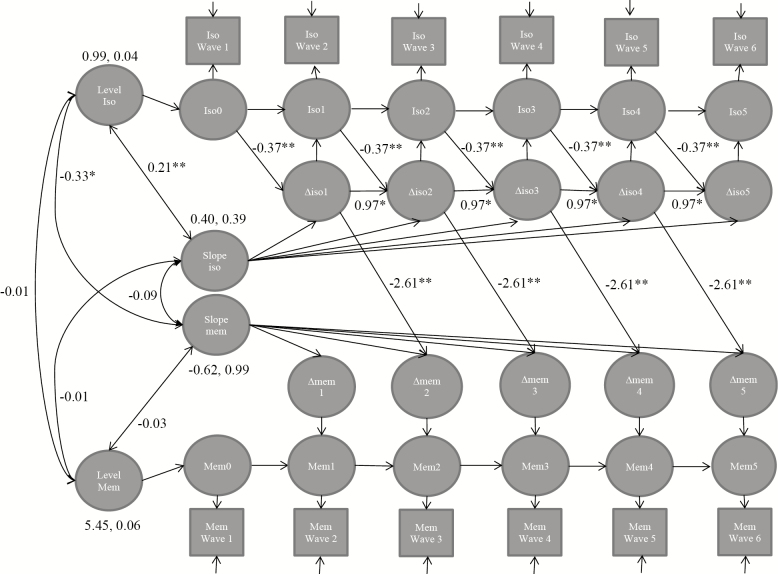
Full bivariate latent change score model for women (*N* = 6,123). The diagram shows that the level and change of social isolation predicts the change in social isolation and the change in social isolation predicts memory over the six waves. Only significant level-to-change and change-to-change paths are shown (see Table 1 for the estimates of all paths), paths with no coefficient are fixed to 1, fully adjusted models. The model fit: −2LL = 176,095.99, *df* = 473, AIC = 353,138, BIC = 356,316. Iso = social isolation, mem = memory, *p < .05, **p < .01, ***p < .001.

In women, estimating the extended LCS effects of previous changes on subsequent changes improved the fit of the models: AIC/BIC values were lower (better) when change-to-change parameters were included in the models (Table 2). Women who had a higher initial level of social isolation were more likely to change to being less isolated over the follow-up (Table 1, Figure 2). Moreover, women who experienced a larger change in isolation were more likely to experience another change in isolation in the subsequent follow-up (Table 1, Figure 2). Women who experienced a larger increase in social isolation had a steeper decrease in memory: one increase in the social isolation scale involved a decrement of 2.6 on the memory score in the subsequent follow-up window, compared to the women who did not experience any loss (Table 1, Figure 2). This would be on average 0.5 points (9%) decrease in memory over a 2-year period. Results from the model comparisons showed that these paths for the level of social isolation to change in social isolation (change-to-change model 1 for women, Table 2), change in social isolation to subsequent change in social isolation (change-to-change model 5 for women, Table 2), and change in social isolation to subsequent change in memory (change-to-change model 7 for women, Table 2) were necessary in the model and cannot be set to 0.

## Discussion

This study investigated the relationship between level and change in social isolation and memory in a nationally representative sample of older people in England. Previous studies have proposed that social isolation results in poorer memory, or alternatively that poorer memory leads to social isolation. To untangle the direction of the effect underlying the association between social isolation and memory, we used extended latent change score models over six measurement occasions spanning 10 years. Results suggest that the association between social isolation and memory decline is driven by the affect of social isolation on memory, rather than the reverse. The results thus lend support to previous findings indicating that social isolation is associated with declines in cognition (Andrew & Rockwood, 2010; Shankar et al., 2013). Unlike these previous studies, we were able to test competing pathways between changes in memory and social isolation.

In terms of the magnitude of the effect, men with a high-social isolation score (4+) experienced a memory decline of 0.9 points (18%) over 2 years, compared with a 0.3-point memory decline (6%) among men with an average isolation score of one. In women, when isolation increased two points, the subsequent memory decline in the next 2 years was on average one point (18%) compared with an average memory decline of 0.2 point (4%) over a 2-year period. These memory changes related to higher or increased social isolation are similar to the rates (12%–30% decline in 2 years) found among older people with progressive memory decline (Cloutier, Chertkow, Kergoat, Gauthier, & Belleville, 2015) and mild cognitive impairment (Petersen et al., 2009) prior to dementia, compared with 2%–4% biannual decline in normal aging.

In this study, analyses were conducted separately for men and women and so differences by gender cannot be formally assessed. However, they are suggestive of gender differences. Thus, although the direction of the effect was the same in men and women, results suggest higher stability and a weaker association between social isolation and memory in men compared with stronger interrelationships between level and change in social isolation and change in memory in women. In women, larger changes in social isolation predicted larger subsequent changes, including a tendency to recover when the isolation reached a higher level, that is, those who had a high level of isolation tended to move toward being less isolated rather than becoming more isolated. Women experiencing increased isolation also showed declines in memory. The finding is partly in line with previous results showing that an increase in new confidantes in social networks is associated with improved functional and self-rated health and decreased depressive symptoms (Cornwell & Laumann, 2015). Although increased isolation seemed to result in memory decline in women, the successive recuperative movement to reduced isolation may in turn alter the pattern of change in memory.

In men, social isolation scores showed little change over time, and the number of men with a high-isolation score was small. There may be several reasons for these suggestions of gender differences. One relevant factor is the inclusion of living alone in the social isolation score we used. Older men in general, are more likely to live with a partner, and apart from this variation in inputs to the scale used, partners may provide access to other social contacts. The difference between the genders may also reflect a different time window of changes: changes in social isolation or replacement of social losses in men may happen faster than can be detected using 2-year repeated measures. For instance, older men are more likely to find a new partner after widowerhood than are women, and tend to do so more quickly (Wu, Schimmele, & Ouellet, 2015). There may also be gender differences in whether older people try to compensate for losses themselves by building new social connections or whether family or friends differentially initiate increased contacts after losses. Further research on unraveling these processes would require more detailed data on who initiates social interactions in older people’s networks.

There are some limitations to the present study. Although the analysis used FIML to take into account attrition over the 10-year period, the sample may be initially selected. Those with more social connections and better memory were more likely to accept the initial invitation to take part. Because the ELSA does not cover people living in institutional settings at the start of the study, results can only be generalized to older people living in the community. Even after using FIML and several covariates known to be associated with both social isolation and memory and their attrition, the possibility of some bias arising from attrition may not be accounted for. Due to complexity of the LSC modeling, gender differences were not formally tested. Social isolation scores can be constructed in different ways which complicates comparison between studies. We chose to use a score used previously in analyses of data from ELSA partly in order to facilitate comparison (de Oliveira et al., 2010; Shankar et al., 2013). This score was designed to identify people with low levels of key interactions and does not capture dimensions related to perceived support or quality of contacts which may also be important. Some previous studies have suggested that using scales with items representing many dimensions may lead to spurious results because of possible offsetting effects (Cornwell & Laumann, 2015; Rafnsson et al., 2017; Shankar et al., 2013). However, the social isolation scale we use may in some cases be insufficient for identifying the level of isolation: some people may live alone, have less than monthly contacts with family and friends and not be a member of an organization but have other types of contact, for example, with neighbors, formal caregivers, or whereas out in the neighborhood carrying out usual tasks (errands, shopping). As already mentioned, we used data collected at two yearly intervals to assess changes in social isolation score, and so may miss changes which occur in shorter time periods. There are also some limitations to some of the other measures used. The measure of physical health, for example, was a binary indicator based on self-reported illness which limited activities and cannot differentiate multimorbidity or the magnitude of limitation. Future studies should investigate further the role of physical health and depression in the association between social isolation and cognition.

The strength of this study is that it uses six measurement occasions to disentangle the associations between the level and change in social isolation and memory over a 10-year period. Social isolation is an easy and economical measure to include in questionnaires and the information required for the scale can be collected from proxies. Although men and women appear to differ in how strong and complex the associations were, the overall message from the models for both genders was that social isolation predicts changes in memory. This is consistent with results from some previous studies which were not able to examine affect of both levels and changes to the same extent as in this analysis (Andrew & Rockwood, 2010; Shankar et al., 2013; Thomas, 2011). Our results revealed an interesting dynamic pattern of change in social isolation among women with a tendency for compensating shifts over time. Overall results indicate that older men with high levels of isolation and women with continuing isolation over time experience memory decline and might benefit from targeted interventions. Further research is needed to formally assess and investigate further gender differences in associations between social isolation and memory decline and to test whether interventions to reduce social isolation would have beneficial effects on memory retention. This is especially important because: firstly family composition and history, such as partnership and number of children is associated with both the frequency of current social contacts (Grundy & Read, 2012) and level of cognition (Read & Grundy, 2017) and secondly cohorts now approaching later life include higher proportions with no children and who live alone.

## Funding

This work was supported by an award from the UK Economic and Social Research Council (1025561/2/3) and from the UK Economic and Social Research Council and National Institute for Health Research (ES/L001896/1)

## Author Contributions

S. Read planned the study, analyzed the data and wrote the first version of the article. A. Comas-Herrera and E. Grundy contributed to writing and revising the article.

## Conflict of interest

None.

## Supplementary Material

gbz152_suppl_Supplementary_Tables
