# Supplementary material for: Social Isolation and Memory Decline in Later-life
Source: J Gerontol B Psychol Sci Soc Sci. 2019 Nov 29;75(2):367–76. doi: 10.1093/geronb/gbz152 (PMC6963696; doi:10.1093/geronb/gbz152)
Supplement: gbz152_suppl_Supplementary_Tables [file gbz152_suppl_supplementary_tables.pdf]

Supplementary Table 1. Distribution of the sample by time-invariant covariates at wave 1

	Men (*n* = 5110)		Women (*n* = 6123)	
Variables	% or mean (SD)	*n*	% or mean (SD)	*n*
Age in years	64.8 (9.87)	5110	65.4 (10.40)	6123
Education		5110		6115
Tertiary	27.8		17.4	
Secondary	22.0		21.6	
Other	14.2		13.4	
No qualification	36.6		47.6	
Net wealth quintile	3.1 (1.43)	5076	2.3 (1.40)	6058
House owner	81.2	5090	78.8	6091
Smoking		5104		6115
Current smoker	17.4		18.1	
Past smoker	56.6		38.3	
Never smoked	26.0		43.6	
Physical activity	2.0 (0.88)	5104	1.9 (0.88)	6114

Supplementary Table 2. Distribution of social isolation, memory and time-varying covariates in men, waves 1- 6 (% or mean (SD) in the adjusted model[a])

Variables	Wave 1 (2002/03) *n* = 5110[b]	Wave 2 (2004/05) *n* = 3973[b]	Wave 3 (2006/07) *n* = 3332[b]	Wave 4 (2008/09) *n* = 2896[b]	Wave 5 (2010/11) *n* = 2722[b]	Wave 6 (2012/13) *n* = 2457[b]	Growth[c]
Social isolation score	1.2 (1.04)	1.2 (0.95)	1.3 (0.97)	1.2 (0.93)	1.4 (0.99)	1.4 (0.98)	m: 0.03***
	n = 4650	*n* = 3456	*n* = 2876	*n* = 2456	*n* = 2395	*n* = 2124	r: 0.01***
Memory score	4.6 (1.77)	4.6 (1.81)	4.6 (1.91)	4.4 (1.89)	4.3 (2.01)	4.2 (2.06)	m: -0.04***
	n = 5015	*n* = 3865	*n* = 3264	*n* = 2804	*n* = 2591	*n* = 2336	r: 0.02***
Depressive symptoms (CES-D8 >2)	20	21	20	20	24	21	m: -0.01
	n = 5015	*n* = 3845	*n* = 3255	*n* = 2793	*n* = 2581	*n* = 2330	r: 0.00
Liming long-term illness	35	38	38	40	42	47	m: 0.03***
	n = 5107	*n* =3914	*n* = 3330	*n* = 2895	*n* = 2719	*n* = 2456	r: 0.004***
Working or doing voluntary work	49	46	41	35	30	26	m: -0.05***
	n = 5108	*n* =3973	*n* = 3332	*n* = 2896	*n* = 2722	*n* = 2457	r: 0.003***
Age among those in the sample	64.8 (9.87)	66.0 (9.51)	67.5 (9.23)	68.8 (8.78)	70.1 (8.29)	71.5 (8.03)	

[a] Adjusted for age, education, net wealth quintile, tenure status, limiting long-term illness, smoking and physical activity. [b] Core sample with full or partial interview. [c] Linear mean growth (m) and residual variance growth (r) in Latent Growth Curve, *$*p < 0.05$, $** p < 0.01$, $***p < 0.001$.*

Supplementary Table 3. Distribution of social isolation, memory and time-varying covariates in women, waves 1- 6 (% or mean (SD) in the adjusted model[a])

Variables	Wave 1 (2002/03) 1 (*n* = 6123)[b]	Wave 2 (2004/05) 2 (*n* = 4799)[b]	Wave 3 (2006/07) 3 (*n* = 4157)[b]	Wave 4 (2008/09) 4 (*n* = 3685)[b]	Wave 5 (2010/11) 5 (*n* = 3480)[b]	Wave 6 (2012/13) 6 (*n* = 3168)[b]	Growth[c]
Social isolation score	1.1 (1.04)	1.2 (1.04)	1.2 (1.03)	1.2 (1.02)	1.4 (1.04)	1.4 (1.06)	m: 0.04***
	n = 5532	*n* =4227	*n* = 3551	*n* = 3112	*n* = 3057	*n* = 2716	r: 0.01***
Memory score	4.8 (1.81)	4.9 (1.84)	4.8 (1.89)	4.7 (1.93)	4.5 (1.95)	4.5 (1.97)	m: -0.06***
	n = 6020	*n* =4753	*n* = 4065	*n* = 3562	*n* = 3328	*n* = 3023	r: 0.02***
Depressive symptoms (CES-D8 >2)	28	28	27	27	27	24	m: 0.01
	n = 6025	*n* =4743	*n* = 4049	*n* = 3551	*n* = 3309	*n* = 3001	r: 0.02***
Liming long-term illness	35	39	41	43	46	48	m: 0.002***
	n = 6117	*n* =4799	*n* = 4155	*n* = 3684	*n* = 3479	*n* = 3165	r: 0.003***
Working or doing voluntary work	42	45	35	30	25	21	m: -0.04***
	n = 6118	*n* =4763	*n* = 4157	*n* = 3685	*n* = 3480	*n* = 3168	r: 0.003***
Age among those in the sample	65.4 (10.40)	66.8 (10.06)	68.4 (9.79)	69.7 (9.26)	70.9 (8.81)	72.5 (8.44)	

[a] Adjusted for age, education, net wealth quintile, tenure status, limiting long-term illness, smoking and physical activity. [b] Core sample with full or partial interview. [c] Linear mean growth (m) and residual variance growth (r) in Latent Growth Curve, $*p < 0.05$, $** p < 0.01$, $***p < 0.001$.

Supplementary Table 4. Unstandardized parameter estimates (Standard Error) for the univariate Latent Change Score models of social isolation and memory in waves 1 – 6 (W1 - W6)

	Social isolation		Memory	
	Men	Women	Men	Women
Intercepts				
Level	1.01 (0.04)***	1.00 (0.03)***	5.08 (0.05)***	5.47 (0.05)***
Growth	0.17 (0.11)	0.42 (0.16)*	-0.43 (0.44)	-1.01 (0.41)*
Residual variances				
Observed variable	0.41 (0.01)***	0.33 (0.01)***	1.26 (0.02)***	1.32 (0.02)***
Level	0.74 (0.02)***	0.63 (0.02)***	0.99 (0.04)***	1.04 (0.05)***
Growth	0.03 (0.02)	0.09 (0.08)	0.02 (0.01)*	0.04 (0.02)*
Association between level and growth	0.07 (0.08)	-0.24 (0.11)*	-0.61 (0.59)	0.48 (0.33)
Proportional change	-0.14 (0.11)	-0.40 (0.17)*	-0.61 (0.59)	-0.46 (0.31)
Change on change	-0.06 (0.24)	1.31 (0.46)**	1.91 (1.27)	1.67 (0.68)*
Effects of time-invariant covariates				
Age on level	0.01 (0.00)***	0.02 (0.01)***	-0.06 (0.00)***	-0.06 (0.00)***
Age on growth	0.01 (0.00)**	0.01 (0.00)**	-0.04 (0.04)	0.03 (0.02)
Education on level (ref=tertiary)				
Secondary	0.11 (0.04)**	0.02 (0.04)	-0.27 (0.05)***	-0.22 (0.05)***
Other	-0.01 (0.05)	-0.06 (0.04)	-0.66 (0.06)***	-0.58 (0.06)***
No qualification	0.21 (0.04)***	0.05 (0.04)	-0.97 (0.05)***	-0.96 (0.05)***
Education on growth (ref=tertiary)				
Secondary	0.03 (0.02)	0.01 (0.00)	-0.16 (0.16)	-0.10 (0.07)
Other	0.04 (0.02)*	-0.02 (0.02)	-0.40 (0.39)	-0.26 (0.18)
No qualification	0.04 (0.03)	0.02 (0.02)	-0.59 (0.57)	-0.43 (0.30)
Wealth on level	-0.04 (0.01)***	-0.07 (0.01)***	0.12 (0.01)***	0.13 (0.01)***
Wealth on growth	-0.01 (0.00)*	-0.03 (0.01)*	0.07 (0.07)	0.06 (0.04)
Home ownership on level	-0.46 (0.05)***	-0.29 (0.04)***	0.12 (0.06)*	0.18 (0.05)***
Home ownership on growth	-0.04 (0.05)	-0.12 (0.05)	0.07 (0.08)	-0.08 (0.06)

Smoking on level (ref=never smoked)				
Current smoker	0.06 (0.05)	0.22 (0.04)***	0.07 (0.04)	0.03 (0.05)
Past smoker	-0.04 (0.04)	0.01 (0.03)	0.11 (0.04)*	0.03 (0.04)
Smoking on growth (ref=never smoked)				
Current smoker	0.08 (0.03)**	0.10 (0.04)**	0.02 (0.05)	0.00 (0.03)
Past smoker	-0.01 (0.01)	0.01 (0.01)	0.06 (0.07)	0.02 (0.02)
Physical activity on level	-0.11 (0.02)***	-0.10 (0.02)***	0.11 (0.03)***	0.17 (0.02)***
Physical activity on growth	-0.02 (0.01)	-0.04 (0.02)*	0.06 (0.07)	0.08 (0.05)*
Time-varying effects				
Limiting long-term illness W1	-0.01 (0.03)	0.06 (0.02)*	-0.16 (0.04)***	-0.04 (0.04)
W2	-0.08 (0.03)**	0.01 (0.02)	-0.02 (0.04)	0.04 (0.04)
W3	-0.03 (0.03)	0.01 (0.02)	0.04 20.05)	0.01 (0.04)
W4	-0.16 (0.03)***	-0.10 (0.02)***	-0.03 (0.05)	-0.03 (0.04)
W5	0.07 (0.03)*	0.02 (0.03)	-0.01 (0.06)	-0.12 (0.05)**
W6	0.10 (0.04)**	0.07 (0.03)*	-0.14 (0.06)*	-0.06 (0.05)
Depression W1	0.02 (0.01)**	0.02 (0.01)**	-0.02 (0.01)	-0.03 (0.01)**
W2	0.04 (0.01)***	0.03 (0.00)***	-0.06 (0.01)***	-0.03 (0.01)**
W3	0.03 (0.01)**	0.04 (0.01)***	-0.06 (0.02)***	-0.03 (0.01)*
W4	0.06 (0.01)***	0.04 (0.01)***	-0.07 (0.01)***	-0.05 (0.01)***
W5	0.04 (0.01)***	0.03 (0.01)***	-0.06 (0.02)***	-0.04 (0.01)***
W6	0.04 (0.01)**	0.04 (0.01)***	-0.05 (0.02)**	-0.04 (0.01)**
Working/voluntary work W1	-0.03 (0.02)	-0.04 (0.02)*	-0.01 (0.04)	-0.11 (0.03)**
W2	0.03 (0.03)	0.00 (0.02)	0.00 (0.04)	0.05 (0.04)
W3	0.01 (0.02)	-0.01 (0.02)	0.10 (0.04)*	0.12 (0.04)**
W4	-0.12 (0.03)***	-0.07 (0.02)**	0.01 (0.04)	0.11 (0.04)**
W5	-0.04 (0.03)	-0.05 (0.02)*	-0.03 (0.05)	0.04 (0.05)
W6	-0.04 (0.03)	-0.01 (0.03)	0.10 (0.06)	0.09 (0.06)

$*p < 0.05$, $**p < 0.01$, $***p < 0.001$
